# Effect of inferior oblique myectomy on ocular torsion according to the absence of the trochlear nerve in unilateral congenital superior oblique palsy

**DOI:** 10.1371/journal.pone.0283555

**Published:** 2023-03-23

**Authors:** Yeji Moon, Byung Joo Lee

**Affiliations:** Department of Ophthalmology, Asan Medical Center, University of Ulsan College of Medicine, Seoul, Republic of Korea; Cairo University Kasr Alainy Faculty of Medicine, EGYPT

## Abstract

**Objectives:**

To evaluate the effect of inferior oblique (IO) myectomy on ocular torsion according to the absence of the trochlear nerve in unilateral congenital superior oblique palsy (UCSOP).

**Methods:**

We retrospectively reviewed the clinical data of patients who had been diagnosed with UCSOP and underwent ipsilateral IO myectomy (n = 43). Patients were classified into the present and absent groups according to the absence of the trochlear nerve and superior oblique hypoplasia on magnetic resonance imaging (MRI). For quantitative analysis of ocular torsion, disc-fovea angles (DFA) were collected in both eyes using fundus photographs taken within three months before surgery and one month after surgery.

**Results:**

DFA of the paretic eye did not differ according to the absence of the trochlear nerve (9.4±5.6° in the present group vs. 11.0±5.4° in the absent group, *p* = 0.508). However, the present group had a larger DFA in the non-paretic eye than the absent group (14.1±6.7° in the present group vs. 8.0±5.0° in the absent group, *p* = 0.003). The change of ocular torsion after IO myectomy in the paretic eye was -5.3±3.7° in the present group and -4.8±3.5° in the absent group, respectively (*p* = 0.801). In the non-paretic eye, the change in DFA was -1.5±3.0° in the present group, which was larger than that in the absent group (0.7±2.6°, *p* = 0.047). In the multivariate analysis, the change in DFA was correlated with only the preoperative DFA (standardized *β* = -0.617, *p*<0.001 in the paretic eye, and standardized *β* = -0.517, *p*<0.001 in the non-paretic eye).

**Conclusions:**

In the paretic eye, there was no significant difference in the change of ocular torsion between both groups, whereas in the non-paretic eye, the present group had a larger change in DFA after IO myectomy than the absent group. However, in the multivariable analysis, the change in ocular torsion was significantly correlated with preoperative excyclotorsion but not with the presence of the trochlear nerve itself.

## Introduction

Technical advancements in magnetic resonance imaging (MRI) have enabled visualization of the trochlear nerve [1]. Accordingly, the absence of the trochlear nerve and superior oblique muscle (SO) hypoplasia, which can be classified as a congenital cranial dysinnervation disorder, has been accepted as one of the pathophysiologies of unilateral congenital superior oblique palsy (UCSOP) [2–4]. Several studies have reported clinical characteristics according to the absence of the trochlear nerve, and Lee et al. revealed that the absence of trochlear nerve can contribute to the amount of ocular torsion in unilateral SOP [5–7].

As the primary action of SO is incyclotorsion, patients with UCSOP present with ocular excyclotorsion. Furthermore, previous studies have shown that excyclotorsion can also be present in non-paretic contralateral eyes [8–10]. To improve ocular torsion in addition to a reduction in vertical deviation, several types of inferior oblique muscle (IO) weakening surgery can be performed [11–16]. Of them, IO myectomy is a simple, safe, and effective surgery [17, 18]. Previously, Lee et al. reported the surgical outcomes in UCSOP after IO myectomy according to the absence of the trochlear nerve [19]. However, the effect of IO myectomy on ocular torsion according to the absence of the trochlear nerve remains unknown.

Therefore, this study aimed to evaluate the effect of IO myectomy on ocular torsion according to the absence of the trochlear nerve in patients with UCSOP. Furthermore, we investigated the correlation between changes in ocular torsion and various clinical factors, including preoperative ocular torsion, vertical deviation, head tilt, IO overaction (IOOA), and SO underaction (SOUA).

## Materials and methods

This study was conducted in accordance with the tenets of the Declaration of Helsinki. The study protocol was approved by the institutional review board of the Asan Medical Center (IRB No. 2022–1117), and the requirement for informed consent was waived owing to the retrospective study design.

### Study subjects

We retrospectively reviewed the medical records of patients who had been diagnosed with UCSOP and underwent ipsilateral IO myectomy between July 2014 and June 2022 at Asan Medical Center. The UCSOP diagnosis was made based on the presence of SOUA (under-depression in adduction) and/or IOOA (overelevation in adduction), positivity in the Parks-Bielschowsky three-step test, large fusional amplitudes of vertical deviation, facial asymmetry, and a reliable history of long-standing strabismus or persistent anomalous head posture.

Preoperative data on the degree of vertical deviation in the primary position, IOOA, and SOUA were obtained. The degree of vertical deviation was measured using the alternate prism cover test. The degree of IOOA was graded between 0 and +4, and the degree of SOUA was between 0 and -4 according to the deviation of the pupil on nasal and inferonasal fixation, respectively. A score of 0 were given for no deviation, and up to 4 points were given at intervals of 0.5 points according to the amount of deviation measured in mm. We also measured the degree of head tilt on the clinical photographs using PentaVision image software (Asan Medical Center, Seoul, Korea) and a built-in protractor. The photograph was taken in a natural sitting position while the patient was fixating on a distant target at 6 m. In the photograph, the degree of head tilt was defined as the angle between the vertical midline and the vertical axis of the face.

MRI scans were obtained using a 3-Tesla system (InteraAchieva; Phillips Healthcare, Best, Netherlands) to investigate the presence of the trochlear nerve. When the trochlear nerve was absent and ipsilateral SO muscle hypoplasia was confirmed, the patient was classified into the absent group. SO muscle hypoplasia was defined as a ratio of the paretic/fellow SO muscle area less than 0.75 [20]. Inversely, when the trochlear nerve was present, and there was no significant asymmetry in either SO muscle, the patient was classified into the present group. When the absence of a trochlear nerve was not in accordance with SO hypoplasia, subjects were excluded from this study.

The following exclusion criteria were applied: (1) any signs of bilateral SOP; (2) any etiologies related to acquired SOP, such as head trauma; (3) concurrent craniosynostosis or muscular torticollis; (4) previous strabismus surgery; and (5) insufficient data for fundus photography and MRI.

### Assessment of ocular torsion

**Fig 1:**
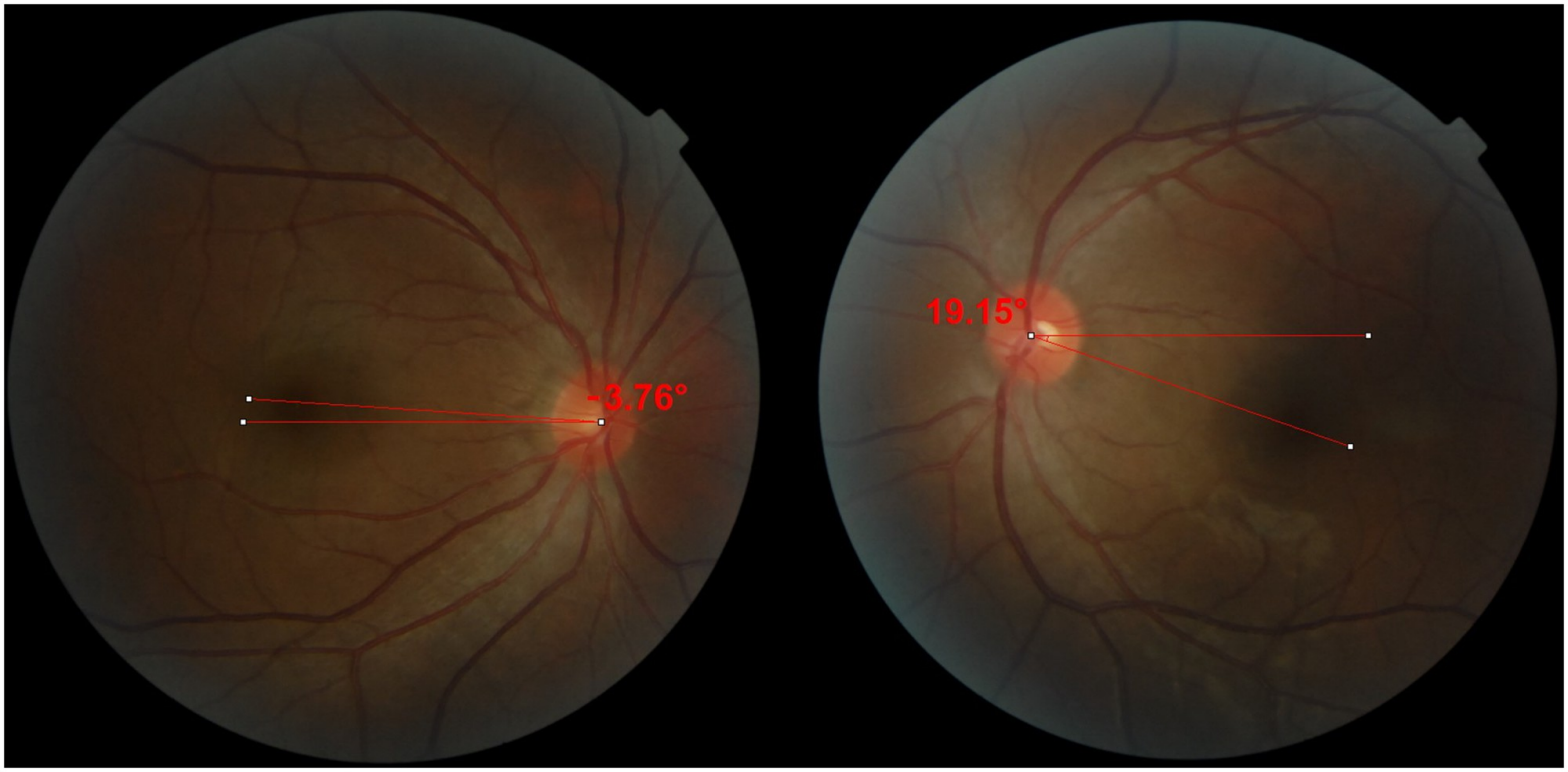
The measurement of the disc-foveal angle (DFA) using fundus photographs. The DFA is -3.76° in the right eye and 19.15° in the left eye.

As described in previous studies, ocular torsion was measured quantitatively using digital fundus photography [9, 21]. The preoperative fundus photograph was taken within three months before surgery. The postoperative fundus photograph was taken one month after surgery to evaluate only the effect induced by the surgery excluding the other factors. Fundus photographs were obtained using TRC-50DX (Topcon Medical System, Tokyo, Japan). Patients were asked to gaze at the internal fixation target to align their eyes at the primary position. The disc-foveal angle (DFA) was measured on a well-focused fundus photograph using the PentaVision image software (Asan Medical Center, Seoul, Korea) and a built-in protractor. DFA was defined as the angle between a horizontal line passing through the geometric center of the optic disc and the line connecting the fovea and the geometric center of the optic disc. (Fig 1) The DFA is expressed as a negative value when the vertical position of the fovea was located above the center of the optic disc, which means incyclotorsion. DFA measurements were independently performed twice by a single examiner (YM), from which the mean values were obtained. The intraclass coefficient of the parameters was 0.981.

### Surgical procedures

All procedures were performed under general anesthesia. A conjunctival incision was made in the inferotemporal quadrant of the bulbar conjunctiva. After dissecting the Tenon’s capsule and isolating the IO muscle, two hemostatic clamps were applied, separated by approximately 6–8 mm. A portion of the IO muscle between clamps was excised. After electrocauterization of the resection margin, the muscle was released, and the proximal part of the muscle was retracted into Tenon’s capsule. The conjunctiva was sutured with 8–0 Vicryl without Tenon’s capsule sutures. No surgical complications were encountered in any of the patients.

### Statistical analysis

Continuous data are presented as the mean ± standard deviation (SD) and range, whereas categorical data are presented as proportions and percentages. The Mann-Whitney U test and Chi-square test were used to compare the clinical characteristics between the absent and present groups. Univariable and multivariable linear regression analyses were performed to identify factors significantly associated with changes in ocular torsion after IO myectomy. Variables with univariate regression and a *p*-value of <0.1 were included in the multivariate regression analysis. Statistical significance was established at a *p*-value of < 0.05 for all calculations. All statistical analyses were performed using SPSS version 23.0 (SPSS, Inc., Chicago, IL, USA).

## Results

### Baseline characteristics according to the absence of the trochlear nerve

A total of 43 patients were included in the final analysis. Of them, 22 patients (51.2%) with confirmed absence of the trochlear nerve and SO hypoplasia on MRI were classified as the absent group, and the other 21 were classified as the present group. Baseline demographic and preoperative data are summarized in Table 1. The mean age at surgery was 27.9 ± 20.7 years, and females accounted for 39.5% of the total patients. There were no significant differences in the degree of vertical deviation, IOOA, or SOUA. However, the absent group showed a larger angle of head tilt to the contralateral side than the present group (7.8 ± 3.6° vs. 4.9 ± 2.8°, *p* = 0.001).

**Table 1:** Baseline demographic and preoperative data of the study population.

Variables	Total (n = 43)	Present group (n = 21)	Absent group (n = 22)	*p*-value
**Age at surgery, years**	27.9 ± 20.7 (4.0–63.3)	28.5 ± 19.8 (4.0–63.3)	27.4 ± 21.9 (4.1–63.3)	0.309
**Sex (male:female)**	26:17	14:7	12:10	0.537
**Operative eye (right:left)**	19:24	11:10	8:14	0.364
**Vertical deviation, prism diopters**	12.2 ± 6.6 (2–35)	13.2 ± 6.1 (5–25)	11.3 ± 7.1 (2–35)	0.126
**Inferior oblique overaction**	2.2 ± 0.8 (0.5–4.0)	2.1 ± 0.7 (1.0–3.0)	2.2 ± 0.9 (0.5–4.0)	0.417
**Superior oblique underaction**	-1.2 ± 0.8 (-3.0–0.0)	-1.2 ± 0.7 (-2.0–0.0)	-1.1 ± 0.9 (-3.0–0.0)	0.354
**Head tilt angle, º**	6.4 ± 3.5 (0.0–16.2)	4.9 ± 2.8 (0.0–11.9)	7.8 ± 3.6 (0.0–16.2)	**0.001**

The *p*-values marked in bold indicate a statistically significant difference between the two groups.

### Comparison of ocular torsion according to the absence of the trochlear nerve

The DFA of the paretic eye did not differ according to the absence of the trochlear nerve. However, the present group had a larger DFA in the non-paretic eye than the absent group (14.1 ± 6.7° vs. 8.0 ± 5.0°, *p* = 0.003). Accordingly, the sum of the DFAs in both eyes was larger in the present group than in the absent group (23.5 ± 9.5° vs. 18.9 ± 6.2°, *p* = 0.041).

After IO myectomy, the DFA of the paretic eye decreased to 4.1 ± 4.3° and 6.1 ± 4.2° in the present and absent groups, respectively, and the postoperative DFA did not differ significantly between the groups (*p* = 0.136). Meanwhile, the DFA of the non-paretic eye decreased to 12.6 ± 5.8° and 8.6 ± 4.9° in the present and absent groups, respectively. After surgery, the difference in the DFA of the non-paretic eye remained statistically significant between the two groups (*p* = 0.047). However, the difference in the sum of DFAs became insignificant after IO myectomy (16.7 ± 7.5° vs 14.8 ± 5.4°, *p* = 0.291) (Table 2).

**Table 2:** Comparison of the disc-fovea angle according to the absence of the trochlear nerve.

	Total (n = 43)	Present group (n = 21)	Absent group (n = 22)	*p*-value
**Preoperative ocular torsion, °**				
** Paretic eye**	10.2 ± 5.5 (-2.9–22.6)	9.4 ± 5.6 (-2.9–21.1)	11.0 ± 5.4 (1.8–22.6)	0.508
** Non-paretic eye**	11.0 ± 6.6 (-4.2–27.5)	14.1 ± 6.7 (2.2–27.5)	8.0 ± 5.0 (-4.2–15.1)	**0.003**
** Sum (Paretic + Non-paretic eye)**	21.2 ± 8.2 (0.9–38.6)	23.5 ± 9.5 (0.9–38.6)	18.9 ± 6.2 (11.1–31.3)	**0.041**
**Postoperative ocular torsion, °**				
** Paretic eye**	5.1 ± 4.3 (-3.8–17.0)	4.1 ± 4.3 (-3.8–13.3)	6.1 ± 4.2 (-0.2–17.0)	0.136
** Non-paretic eye**	10.6 ± 5.7 (-2.8–23.9)	12.6 ± 5.8 (3.7–23.9)	8.6 ± 4.9 (-2.8–20.3)	**0.047**
** Sum (Paretic + Non-paretic eye)**	15.7 ± 6.5 (0.9–29.4)	16.7 ± 7.5 (0.9–29.4)	14.8 ± 5.4 (6.7–24.9)	0.291

*p*-values marked in bold indicate statistically significant differences between the two groups.

**Fig 2:**
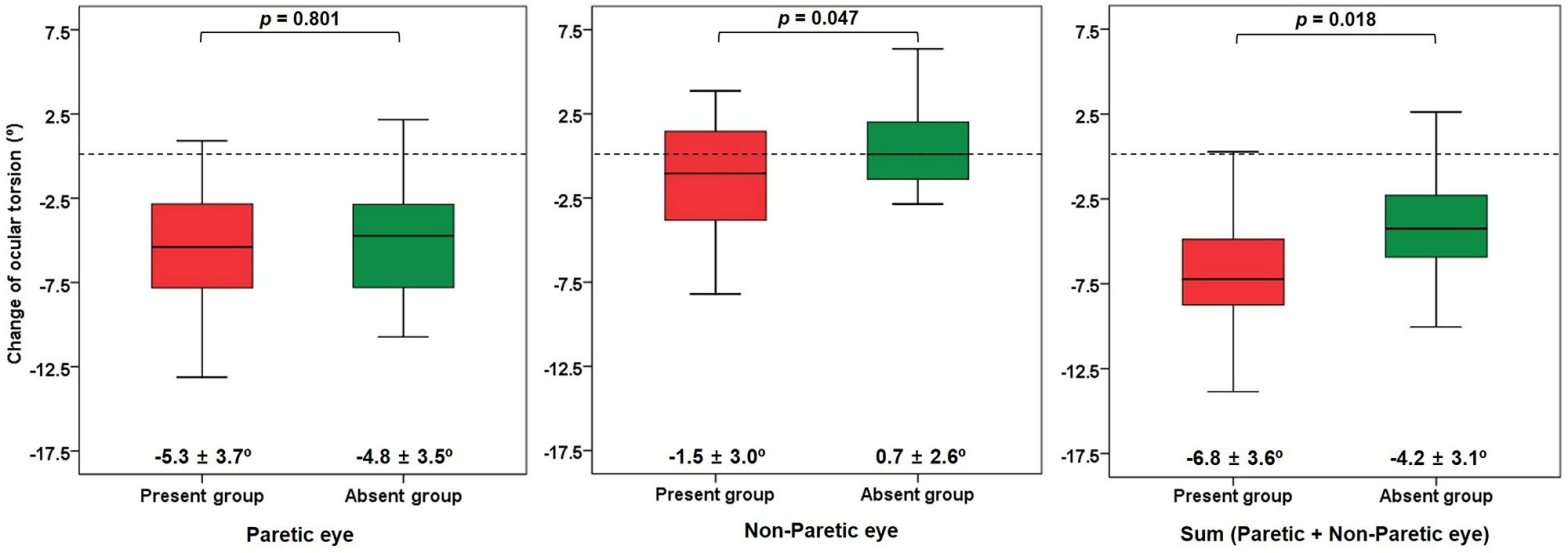
The change of ocular torsion in the paretic and non-paretic eyes according to the absence of the trochlear nerve.

The changes in ocular torsion after IO myectomy are shown in Fig 2. In the paretic eye, the present group showed a decrease in the DFA by 5.3 ± 3.7° and the absent group by 4.8 ± 3.5°, which were significant postoperative changes in both groups (all *p* < 0.001). However, the change in DFA did not differ between the groups (*p* = 0.801). In the non-paretic eye, the change in DFA was -1.5 ± 3.0° in the present group, which was a significant decrease (*p* = 0.032). In contrast, in the absent group, the change in DFA in the non-paretic eye was 0.7 ± 2.6°, and there was no significant change between the preoperative and postoperative DFA (*p* = 0.508). The change in the DFA in the non-paretic eye varied significantly according to the absence of the trochlear nerve (*p* = 0.047). Accordingly, the decrease in the sum of DFAs was larger in the present group than in the absent group (-6.8 ± 3.6º vs. -4.2 ± 3.1°, *p* = 0.018).

### Factors associated with the change of ocular torsion

Table 3 summarizes the univariate and multivariate linear regression analyses for postoperative changes in DFA. The change in DFA in the paretic eye was correlated with the preoperative DFA (standardized *β* = -0.617, *p* < 0.001), which means that the more excyclotorted the paretic eye before surgery, the more the DFA decreased after IO myectomy. However, other clinical factors, including the degree of IOOA, SOUA, and head tilt, were not associated with changes in the DFA in the paretic eye.

**Table 3:** Factors associated with the change of the disc-fovea angle after inferior oblique myectomy (A) in the paretic eye, (B) in the non-paretic eye, and (C) in the sum of both eyes.

**(A)**				
**Variables**	**Univariate**		**Multivariate**	
	**Standardized *β***	***p*-value**	**Standardized *β***	***p*-value**
**Age at surgery**	-0.297	0.054		0.791
**Sex (female)**	-0.002	0.990		
**Group (Absent group)**	0.066	0.673		
**Vertical deviation**	0.065	0.681		
**Inferior oblique overaction**	0.108	0.490		
**Superior oblique underaction**	0.097	0.537		
**Head tilt angle**	0.064	0.683		
**Preoperative disc-fovea angle**	-0.617	**< 0.001**	-0.617	**< 0.001**
**(B)**				
**Variables**	**Univariate**		**Multivariate**	
	**Standardized *β***	***p*-value**	**Standardized *β***	***p*-value**
**Age at surgery**	0.009	0.956		
**Sex (female)**	0.192	0.217		
**Group (Absent group)**	0.364	**0.016**		0.160
**Vertical deviation**	-0.164	0.294		
**Inferior oblique overaction**	0.016	0.919		
**Superior oblique underaction**	0.032	0.839		
**Head tilt angle**	0.252	0.103		
**Preoperative disc-fovea angle**	-0.517	**< 0.001**	-0.517	**< 0.001**
**(C)**				
**Variables**	**Univariate**		**Multivariate**	
	**Standardized *β***	***p*-value**	**Standardized *β***	***p*-value**
**Age at surgery**	-0.286	0.063		0.614
**Sex (female)**	0.156	0.319		
**Group (Absent group)**	0.364	**0.016**		0.111
**Vertical deviation**	-0.071	0.653		
**Inferior oblique overaction**	0.120	0.445		
**Superior oblique underaction**	0.122	0.435		
**Head tilt angle**	0.270	0.080		0.488
**Preoperative disc-fovea angle**	-0.647	**< 0.001**	-0.647	**< 0.001**

In the univariable regression analysis for the change in DFA in the non-paretic eye, the presence of the trochlear nerve and the preoperative DFA were correlated with the change in DFA. Patients with the trochlear nerve or more excyclotorted non-paretic eye had more decrease in DFA in the non-paretic eye after the surgery. However, in the multivariate regression analysis, only preoperative DFA was associated with postoperative changes in the DFA. Accordingly, the sum of ocular torsion changes in both eyes correlated with the sum of preoperative ocular torsion.

## Discussion

The present study investigated the effect of IO myectomy on ocular torsion in patients with UCSOP based on the absence of the trochlear nerve. In the paretic eye, there was no significant difference in the preoperative ocular torsion and the change in ocular torsion between both groups, whereas in the non-paretic eye, the present group had a larger preoperative excyclotorsion and a larger change in DFA after IO myectomy than the absent group. However, in the multivariable analysis, the change in ocular torsion was significantly correlated with preoperative excyclotorsion but not with the presence of the trochlear nerve itself.

After IO myectomy, excyclotorsion in the paretic eye decreased in both groups, and there was no significant difference in the change in ocular torsion between the absent and present groups. Interestingly, in the present group, there was a significant decrease in the excyclotorsion of the non-paretic eye, although it was smaller than that of the paretic eye (-1.5 ± 3.0° vs. -5.3 ± 3.7°, *p* = 0.006). In contrast, there was no significant change in the DFA of the non-paretic eye after surgery in the absent group. In patients with UCSOP, excyclotorsion in the non-paretic eye as well as the paretic eye has been investigated in several previous studies, suggesting that ocular dominance, cyclofusion, or neural adaptation can cause excyclotorsion in the non-paretic eye [9, 22, 23]. According to Hering’s law, conjugate eye movement can induce excyclotorsion of the non-paretic eye in UCSOP. Conversely, a decrease in excyclotorsion in the non-paretic eye can be induced by IO myectomy in the paretic eye. Therefore, we should also focus on the sum of the DFA and its change, not only the DFA of the paretic eye, to evaluate the effect of IO myectomy on ocular torsion. Finally, we found that the effect of IO myectomy on ocular torsion was greater in the present group than in the absent group.

However, in the multivariable regression analysis, it was the preoperative excyclotorsion, not the presence of the trochlear nerve itself, that was correlated with the effect of the IO myectomy on ocular torsion. Considering the self-adjusting effect of IO myectomy [24–26], it was not surprising that the amount of preoperative excyclotorsion was positively correlated with the change in ocular torsion after surgery. Our findings demonstrate that IO myectomy has a self-adjusting effect on ocular torsion as well as on vertical deviation, regardless of the presence of the trochlear nerve.

It should be noted that the excyclotorsion of the non-paretic eye was larger in the present group than in the absent group. Lee et al. previously observed that the “net” incyclotorsion in the paretic eye was more frequent in the absent group than in the present group, suggesting that excyclotorsion in the non-paretic eye was more pronounced in the present group [7]. In this study, we also found a larger excyclotorsion of the non-paretic eye in the present group. This suggests that clinicians should check preoperative ocular torsion in the non-paretic eye, not only in the paretic eye, which can predict the change of ocular torsion after the surgery.

Furthermore, the most important thing that the results of this study suggest is that the analysis of pre- and postoperative ocular torsion according to the absence of the trochlear nerve can provide insight into the mechanics of extraocular muscles in UCSOP patients. Although all patients in both groups were clinically diagnosed with cyclovertical strabismus, termed “UCSOP,” and the clinical features were similar between both groups, except for the excyclotorsion of the non-paretic eye and the degree of head tilt, it can be concluded that the anatomy of the SO was different between both groups. Accordingly, different mechanics of the extraocular muscle may contribute to cyclovertical deviation according to the absence of the trochlear nerve. In the absent group, abnormal contractility of the SO is the main cause of this incomitant strabismus [27]. Meanwhile, in the present group, several factors have been suggested as possible causes, such as rectus pulley displacement [28–30]. and IO tightness [31], which implies that the “UCSOP” in the present group would not be caused by actual SO palsy or paresis. In other words, the different mechanics of the extraocular muscle result in a difference in the amount of ocular torsion, especially in the non-paretic eye, and ultimately a difference in the response to IO myectomy. Further research should be conducted to understand the pathophysiology and refine the mechanics of UCSOP in the present study.

This study has several limitations. First, owing to the retrospective nature of the study, we were unable to obtain data on potential confounding factors, such as ocular dominance. However, the frequency of paretic eye fixation did not differ according to the absence of the trochlear nerve [6]. Therefore, this had little effect on the results. Second, the sample size was relatively small. Also, it should be noted that all patients in this study were aged ≧ two years old. In this study, we included only the patients whose presence or absence of the trochlear nerve could be confirmed using high-resolution MRI, who had undergone IO myectomy in a single center, and whose ocular torsion could be evaluated pre- and postoperatively using fundus photo. Therefore, only a relatively small number of 43 patients were included. Conversely, however, these inclusion criteria have strengthened the accuracy and reliability of the data. Last, postoperative fundus photographs were taken one month after surgery, so longitudinal changes cannot be confirmed. Arici and Oguz reported that the postoperative ocular torsion reduction may be temporary [14]. In contrast, Lee et al. reported postoperative changes in ocular torsion on average one year after surgery, which were similar to those in our study. Therefore, long-term postoperative changes in ocular torsion should be confirmed in future studies.

## Conclusions

In paretic eyes, there was no significant difference in the change in ocular torsion between the absent and present groups. Meanwhile, in the non-paretic eyes, the present group had a larger change in ocular torsion after IO myectomy than the absent group. The change in ocular torsion was significantly correlated with the amount of preoperative excyclotorsion but not with the presence of the trochlear nerve. Therefore, the evaluation of preoperative ocular torsion can help predict changes in ocular torsion after IO myectomy.

## Supporting information

S1 File (XLSX)
